# First Global Blood & Marrow Transplant [GlobalBMT] Conference in Kathmandu with experiences from Nepal, India, Singapore and Sri Lanka

**DOI:** 10.7189/jogh.08.010204

**Published:** 2018-06

**Authors:** Damiano Rondelli, Tan Lip Kun, Vikram Mathews, Lallindra V Gooneratne, Sampurna Tuladhar, Samir Neupane, Santosh Kumar Devadas, Vikas Dua, Bishesh Sharma Poudyal

**Affiliations:** 1 Division of Hematology/Oncology & Center for Global Health, University of Illinois at Chicago, Chicago, Illinois, USA; 2 National University Cancer Institute, Singapore; 3 Department of Haematology, Christian Medical College, Vellore, India; 4 Department of Pathology, University of Colombo, Colombo, Sri Lanka; 5 Civil Service Hospital, Kathmandu, Nepal; ^6^Medical Oncology and Bone Marrow Transplant, Ramaiah University, Bangalore, India ^7^Pediatric Haematology-Oncology and Bone Marrow Transplant, Fortis Hospital, Gurgaon, India

The First GlobalBMT Conference organized on November 5, 2017 in Kathmandu fulfilled one of the educational goals of the active collaboration between the University of Illinois at Chicago Center for Global Health and Civil Service Hospital in Kathmandu (Nepal). This collaboration, that over the years has facilitated the establishment of the first BMT Center in Nepal, is aimed at improving the care of hematologic patients in this country, building capacity in BMT, as well as collaborating on projects related to cost-effectiveness and affordability of BMT in low-middle-income countries (LMICs). To this purpose, the main objective of this first GlobalBMT Conference was to gather physicians from BMT centers in South-East Asia to discuss relevant topics in BMT from the perspective of this part of the world and to find common views on standard practices and specific needs.

The speakers who accepted the invitation to participate in this conference were BMT physicians from Vellore, Bengaluru and New Delhi (India), Singapore (Singapore), Colombo (Sri Lanka), Kathmandu (Nepal) and Chicago (USA). The conference was organized to have presentations focused on the challenges of BMT in LMIC and to generate discussion. Eight lectures were presented and addressed: management of infectious complications in BMT, challenges of graft-vs-host disease (GVHD) in LMIC, challenges in establishing a BMT center in a LMIC, use of post-transplant high dose cyclophosphamide in BMT, methods and quality of stem cell collection and cryopreservation, role of autologous BMT in Non-Hodgkin’s lymphoma, cost-effectiveness and outcome of BMT vs new therapies in multiple myeloma, challenges of pediatric BMT. After the presentations on these general transplant topics, specific experiences and common practices were discussed as key elements in the management of BMT in LMICs. The following represent shared views of the participants on relevant topics that characterize BMT in LMICs.

**1) Challenges of diagnosing infectious diseases in BMT patients:**
- The lack of formal training in Infectious Diseases (ID) in LMICs poses a risk to many patients undergoing BMT. The experience of the BMT Center in Singapore initially was to find internal medicine doctors interested in ID for BMT patients, and establish weekly meetings with BMT staff to discuss each case. Over time this generated more interest in ID, and particularly in transplant ID topics, and it helped the development of a formal ID training (fellowship). This is not yet the case in India, Nepal or Sri Lanka where BMT physicians are for the most part in charge to manage infections on their own.
- A consensus was achieved among participants on recognizing the value of screening and monitoring LMIC BMT patients for tuberculosis (TB). However, a consensus was not achieved on the benefit of a specific pre-emptive antibiotic therapy at the time of transplant since previous findings suggested that the actual overall incidence of TB infections during BMT could be low [1,2].
- Fungal infections are a common cause of morbidity and mortality in LMIC patients because of the environmental conditions and the high cost of standard antifungal treatments [1,3]. Recent data suggested that testing patients’ serum for galactomannan can be a cost effective screening tool to guide therapy for aspergillosis [4], particularly in centers where new antifungal agents, such as posaconazole commonly used in prophylaxis in the US, are not available or not affordable to patients in LMICs.

**2) Challenges of creating a new BMT Center in LMICs:**
- Many of the BMT centers in South East Asia LMICs are relatively new [5-9] and have encountered similar obstacles, such as: limited training of staff, cost of adequate hospital infrastructure, lack of standard procedures, high cost to the patients. Corporate investments in private structures seeking a profit, or in other cases public investments of government hospitals recognizing the cost-effectiveness to cure more patients and preventing their outmigration with secondary poverty of many families, have led to opening new BMT centers in LMICs. However, these efforts can lack adequate quality standards and collabortation with international partners are important in starting new programs.
- Since a major challenge in each LMIC country is the unregulated cost of drugs, which represents a significant burden to the majority of patients and their families, it was agreed, based on the experience in Sri Lanka, that it will be useful to establish policies and regulations for the approval of new drugs in each country. In fact, based on Sri Lanka experience, it is expected that in many LMIC only approved drugs may be available at lower prices.

**3) Challenges of GVHD and rejection in LMICs:**
- In India, GVHD represents a common cause of death among patients receiving an allogeneic BMT [9]. The diagnosis of GVHD is mostly based on clinical observation by BMT physicians with limited time because they care also for many non-transplant patients and cannot implement dedicated GVHD protocols. In addition treatment of GVHD is limited by the scarce availability of immunosuppressive agents and high cost.
- Thalassemia represents a common indication for BMT in South East Asia [5,9-11]. In non-malignant diseases such as thalassemia or aplastic anemia, the use of reduced intensity transplant regimens to lower toxicity, or the use of anti-thymocyte globulin (ATG) or alemtuzumab to reduce the risk of GVHD, leads to a higher risk of rejection, especially when utilizing bone marrow as a source of donor stem cells. For this reason, based on recent findings from an Indian center, it was agreed that it would be beneficial to perform matched related stem cell transplant using donor peripheral blood stem cells (PBSC) and adding post-transplant cyclophosphamide to prevent GVHD [12].
- BMT from haploidentical donors has been increasing in LMICs [13,14] because of much lower cost compared to transplant from cord blood or matched unrelated donors. However, in LMICs there is an unmet need for standardization of indications, donor selection, clinical protocols, as well as data collection, especially for centers not reporting their results to the Center for International Blood and Marrow Transplant Research (CIBMTR).

**4) Autologous stem cell transplantation in myeloma and lymphoma patients:**
- The greatest barrier to this procedure in LMICs is the cost that patients need to afford. There was consensus among participants that it is cost effective to adopt stem cell mobilization strategies that may result in one-day collection. This is more frequently obtained by using chemotherapy-based mobilizing regimens. However, in countries where a generic formulation of the CXCR4 inhibitor plerixafor is available, the use of one dose of this drug in addition to G-CSF the day prior to the collection can be more cost-effective than using chemotherapy and G-CSF [13].
- Stem cell cryopreservation is not always available in LMICs and some centers can only infuse autologous fresh stem cells [15,16]. This requires conditioning regimens lasting for only two days and starting immediately after stem cell collection. Many LMIC centers do not have facilities for controlled rate freezing and regular access to liquid nitrogen and hence use dump freezing at -80 C, which was previously found to be cost effective [17]. This procedure allows the cells to be stored in the lab for up to 6 months. Nevertheless, there has not been any prospective study in LMICs correlating the quality of the stem cell product and the engraftment of patients. A few centers, including Singapore, a couple in India and the Nepal program have implemented stem cell cryopreservation systems with 10% dimethyl sulphoxide (DMSO) and storage in tanks with liquid nitrogen.
- In multiple myeloma patients, there was a consensus on the cost-effectiveness of high dose melphalan followed by autologous BMT [18] and it was emphasized the very limited availability of standard drugs, such as bortezomib and lenalidomide, in many LMICs.

## CONCLUSIONS

### Proposed goals of a GlobalBMT Network in LMICs

- One major limitation for LMIC patients to receive a BMT is the high cost. Some BMT centers in India and Nepal have been able to offer BMT at low cost, and to subsidize patients with limited financial resources by re-investing the profit generated with patients who can pay for BMT. However, it is necessary that BMT centers in LMICs assess, accurately self-report and audit their outcomes, in order to establish if a center can provide a low cost transplant while guaranteeing safety and effectiveness. Reporting to CIBMTR is highly recommended, although it is conceivable that it may require additional costs to the center.

**Figure:**
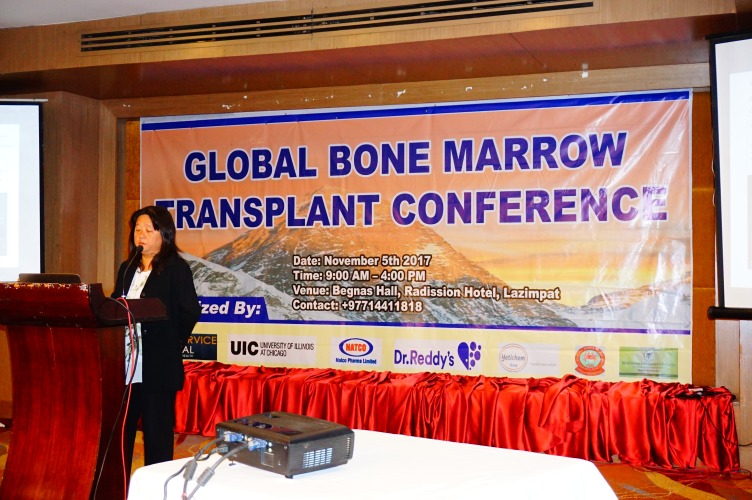
Photo: Dr Tan Lip Kun from Singapore speaking at the GlobalBMT Conference in Kathmandu (Nepal) in November 2017. Photo by Damiano Rondelli (used with permission).
- With a rapidly growing number of BMT centers and growing volume of transplants in LMICs, it seems extremely important that LMIC centers develop infrastructures that support a continuous medical education in BMT, and a quality management program that ensures that deficiencies are identified and corrected. It is also important to encourage LMIC BMT centers to work closely with accrediting agencies in Europe or in the US to establish a step-by-step process that will lead them to receive an international accreditation based on standard procedures and results, matching international benchmarks.
- In order to facilitate the development of LMIC BMT centers with the characteristics described above, the participants agreed that it will be useful to explore possible collaboration through a GlobalBMT Network. This could operate in advising, sharing procedures and policies, providing education tools, and finally coordinating prospective clinical trials validating treatment or diagnostic strategies relevant to LMICs.
